# Effects of sleep deprivation and 4‐7‐8 breathing control on heart rate variability, blood pressure, blood glucose, and endothelial function in healthy young adults

**DOI:** 10.14814/phy2.15389

**Published:** 2022-07-13

**Authors:** Jaruwan Vierra, Orachorn Boonla, Piyapong Prasertsri

**Affiliations:** ^1^ Faculty of Allied Health Sciences Burapha University Chonburi Thailand; ^2^ Exercise and Nutrition Innovation and Sciences Research Unit Burapha University Chonburi Thailand

**Keywords:** autonomic nervous system, blood sugar, breathing exercise, endothelium, sleep

## Abstract

This study investigated the effects of sleep deprivation on heart rate variability (HRV), blood pressure (BP), fasting blood glucose (FBG), and endothelial function as well as the immediate effects of 4‐7‐8 breathing control on HRV and BP. In total, 43 healthy participants aged 19–25 years were classified into two groups: Twenty two in the with sleep deprivation group and 21 in the without sleep deprivation (control) group. Resting heart rate (HR), BP, HRV, FBG, and endothelial function were examined. Subsequently, participants practiced 4‐7‐8 breathing control for six cycles/set for three sets interspersed between each set by 1‐min normal breathing. Thereafter, the HR, BP, and HRV were immediately examined. The HRV, HR, and BP variables and FBG were not significantly different between the two groups. However, endothelial function was significantly lower in the sleep deprivation group than that in the control group (*p* < 0.05). In response to 4‐7‐8 breathing control, low‐ and very‐low‐frequency powers significantly decreased (*p* < 0.05), whereas high‐frequency power significantly increased (*p* < 0.05) in the control group. Moreover, time domain, total power, and very‐low‐frequency power significantly decreased (*p* < 0.05) in the sleep deprivation group. Both groups had significantly decreased HR and systolic BP (*p* < 0.05). HRV, HR, and BP variables showed no significant differences between the groups. Healthy young adults with and without sleep deprivation may have similar HRV, BP, and FBG values. However, sleep deprivation may cause decreased endothelial function. Furthermore, 4‐7‐8 breathing control can help participants improve their HRV and BP, particularly in those without sleep deprivation.

## Graphical abstract

Sleep deprivation may disturb heart rate variability, blood pressure, blood glucose, and endothelial function in healthy young adults. The 4‐7‐8 breathing control may improve these outcomes in those with and without sleep deprivation.

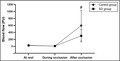

## 1 INTRODUCTION

The National Sleep Foundation (NSF) advises healthy adults aged 18–25 years to sleep for 7–9 h/night (Hirshkowitz et al., 2015). Sleep has two dimensions: Duration (quantity) and depth (quality). When individuals fail to obtain adequate duration or quality of sleep, daytime alertness, and function may decline in response to sleep deprivation (Chattu et al., 2018). Sleep deprivation threatens the health and quality of life of approximately 45% of the world's population (Wade et al., 2008). The American Sleep Association found that 35.3% of adults have less than 7 h of sleep/night and that 50–70 million adults in the US are affected by sleep disorder (Brice et al., 2020). Sleep deprivation can induce several pathophysiological conditions, such as sympathetic overactivity, inflammation, oxidative stress, insulin resistance, and atherosclerosis (Tobaldini et al., 2019). These conditions may further lead to serious health problems, including high blood pressure (BP), diabetes mellitus, stroke, and metabolic disorders (Libianto et al., 2018).

Heart rate variability (HRV), which is regulated by cardiac autonomic nervous system's activity, is significantly reduced after a period of sleep deprivation (Bourdillon et al., 2021). Sleep deprivation also elevates BP through sympathetic overactivity, sympathovagal imbalance, and arterial baroreflex (Virtanen et al., 2015). Moreover, sleep deprivation induces the production of inflammatory mediators by activating the sympathetic system and increasing the oxidative stress reactions, which promote endothelial dysfunction and cardiovascular disease development (Holmer et al., 2021). Sleep deprivation not only causes BP dysregulation but also disrupts the metabolism of glucose and lipids (Liew and Aung, 2021). Deng et al. (2017) found that sleep deprivation is associated with elevation in fasting blood glucose (FBG), BP, and triglycerides and a reduction in high‐density lipoprotein cholesterol. Moreover, glucose tolerance and insulin sensitivity are reduced in people who sleep for only 4–5 h/night (So‐Ngern et al., 2019).

Breathing control is a technique for controlling both the pattern and depth of breathing while promoting upper chest exercise and shoulder relaxation (Solomen and Aaron, 2015). Slow and deep breathing increases the parasympathetic activity, which signals the brain to calm the body down and manages the body's response to anxiety (Jerath et al., 2006; Magnon et al., 2021; Russo et al., 2017). Furthermore, breathing control at 6 breaths/min increases baroreflex sensitivity and reduces the sympathetic activity (Joseph et al., 2005). Similarly, Mason et al. reported that slow breathing at 6 breaths/min increases oxygen saturation, reduces chemoreflex sensitivity, and improves baroreflex sensitivity, which are associated with reduced BP (Mason et al., 2013).

Breathing control for relaxation has several techniques. One is 4‐7‐8 breathing control, a breathing pattern developed by an American physician named Weil A. Inhaling, holding breath, and exhaling for a count of 4, 7, and 8, respectively, is the 4‐7‐8 method of breathing control. The 4‐7‐8 breathing control, which is based on an ancient yogic technique called pranayama, aims at reducing anxiety and facilitating easier sleep (Russo et al., 2017). Short‐term slow breathing reduces oxygen consumption, HR, and BP, increases the amplitude of theta and delta waves (which indicate predominant parasympathetic tone), decreases the sympathetic activity, and improves the sympathovagal balance (Chinagudi et al., 2014). Furthermore, long‐term slow breathing reduces the risk of developing cardiovascular disease and type 2 diabetes mellitus and improves pulmonary function (Jerath et al., 2006; Russo et al., 2017). Accordingly, this study aimed to investigate the effects of sleep deprivation on HRV, BP, FBG, and endothelial function and to identify the immediate effects of the 4‐7‐8 breathing control on HRV and BP in healthy young adults.

## 2 MATERIALS AND METHODS

### 2.1 Study population

Between July and August 2020, 44 young adults aged 19–25 participated in this study. According to a study by Raghul et al. (2018), sample size was calculated by comparing the mean read‐outs of two groups of participants using a statistical formula. Based on a 10.16 ms increase in the standard deviation of normal beat‐to‐normal beat intervals (SDNN) after yogic relaxation (Shavasana), a standard deviation of 9.46, α error of 0.05, and β error of 0.20, the proposed sample size was calculated. It was 18 participants per group with a total of 44 participants, including a dropout rate of 20% (i.e., 4 participants per group). Forty‐four participants were classified into two groups according to sleep duration: Sleep deprivation group (*n* = 22, age: 20.91 ± 1.23 years) and adequate sleep or control group (*n* = 22, age: 22.41 ± 1.50 years). Sleep duration for both the groups was categorized according to the NSF guidelines, which advise 7–9 h of sleep per night for the adults (Hirshkowitz et al., 2015). Hence, the sleep deprivation group slept for less than 7 h/night regularly, whereas the adequate sleep or control group slept for more than 7 h/night.

The Participant's Health Screening Questionnaire was used to assess sleep duration per night (the total amount of time a participant spends asleep) and habitual sleep accumulation (how long that sleep duration has been sustained)—that is, for months or years—during the screening test. The Insomnia Severity Index Questionnaire was also used to assess the sleep quality. Participants were included in the study primarily based on their quantity of sleep. Regarding chronic sleep deprivation, the inclusion criteria, which were based on the American Academy of Sleep Medicine, required participants' sleep disturbances to last for at least 3 months. Additionally, the Participant's Health Screening Questionnaire was used to evaluate underlying diseases, treatment drugs, and illness history. The Thai General Health Questionnaire‐12 was used to evaluate mental health. Moreover, vital signs, including HR, BP, body temperature, and respiratory rate, were measured. To avoid confounding factors that could influence the main results and to ensure that the results mainly arose from the effect of sleep duration, we excluded participants who regularly smoked or drank; had diabetes mellitus, thyroid disease, cardiovascular disease, renal disease, or obesity; were currently on medication for depression or psychiatric disorders; or regularly practiced meditation or breathing control.

The participants were informed of their role in the study both verbally and in writing before providing the consent form for participation. The consent form and the study protocols conformed to the ethical standards of the Human Ethics Committee of Burapha University (Approval No.: G‐SH 028/2563, Date of Approval: July 17, 2020), and of the 1964 Declaration of Helsinki and its later amendments.

### 2.2 Study protocol and 4‐7‐8 breathing control

Participants who passed the criteria and the screening test were asked to keep a consistent bed activity and wake time as usual and requested to come to the laboratory in the morning after 8‐h overnight fasting. Resting HR, BP, HRV, FBG, and endothelial function were then examined. Subsequently, a researcher taught and demonstrated to them the 4‐7‐8 breathing control until they were able to comprehend it well. They were then asked to practice the 4‐7‐8 breathing control. To practice this technique, the participants lay down in a supine position, with their eyes closed lightly and the arms and legs extended fully in a calm and quiet room. One cycle of the 4‐7‐8 breathing control included the following steps: (1) Let the lip part make a whooshing sound and exhale completely through the mouth; (2) close the lips, inhaling silently through the nose, count 1–4 in mind, and then hold breath for 7 seconds; and (3) make another whooshing, exhaling from the mouth, and count 1–8 in mind. They were asked to repeat the 4‐7‐8 breathing control for six cycles/set, for three sets interspersed between each set by 1‐min normal breathing. Finally, their posture was retained for 10 min further to measure their HR, BP, and HRV following the breathing control intervention.

### 2.3 HR and BP assessments

After resting for 10 min and after the 4‐7‐8 breathing control intervention, HR and BP were assessed in the supine position using a digital automatic BP monitor (Microlife BP 3AQ1), which then provided the HR value. The BP cuff was thoroughly enveloped around the arm. The inferior edge of the cuff was nearly 1 inch above the bend of the elbow. We assessed the HR and BP twice with 1 min apart. The mean of the two readings served as the systolic and diastolic BP (SBP and DBP) and HR values. Pulse pressure (PP: SBP − DBP), mean arterial pressure (MAP: DBP + [PP/3]), and rate–pressure product (RPP: HR × SBP) were calculated based on the SBP, DBP, and HR values.

### 2.4 HRV assessment

Following the HR and BP assessments, we assessed the HRV for 10 min. The HRV data were achieved by lead II electrocardiography (PowerLab 4/30, AD Instruments) and analyzed by the HRV module (LabChart® Pro, AD Instruments). HRV variables included the time and frequency domains. The time‐domain consisted of the values of SDNN and the root mean square of successive R‐R interval differences (RMSSD). The frequency‐domain was analyzed using the values of total power, very‐low‐frequency power (direct current potential: −0.04 Hz), low‐frequency power (direct current potential: 0.04–0.15 Hz), high‐frequency power (direct current potential: 0.15–0.4 Hz), and low‐frequency to high‐frequency ratio. Sympathetic and parasympathetic activities, as well as baroreceptor activity, are all described by HRV data. Low‐frequency power, for example, reflects sympathetic activity most of the time, whereas high‐frequency power reflects parasympathetic activity, and the low‐frequency to high‐frequency ratio reflects a balance of sympatho‐vagal activity (Shaffer and Ginsberg, 2017).

### 2.5 FBG assessment

After 8‐h overnight fasting, capillary blood glucose was assessed using a blood glucose monitoring system (Accu‐Chek® Guide, Roche Diabetes Care Inc.), which consisted of lancets, test strips, and a glucometer. After lancing a participant's fingertip, we extracted a drop of capillary blood (approximately 0.6 μl), which was then collected by the test strip inserted into the glucometer. In a few seconds, the screen reported the blood glucose value in mg/dl unit.

### 2.6 Endothelial function assessment

Endothelial function was assessed using the endothelium‐dependent vasodilation technique. Forearm blood flow was measured using the Laser Doppler Flowmetry Module (LDF100C; BIOPAC Systems Inc.), whose probe was attached to the forearm skin in a perpendicular direction. To measure forearm blood flow during occlusion and recovery, a cuff from a standard mercury sphygmomanometer (ABN™, Healthcare Systems) was closely enclosed around the arm proximally to the probe. We inflated the cuff to gain suprasystolic pressure at approximately 200 mm Hg and to completely occlude the brachial artery. Subsequently, the cuff pressure was gradually released. The forearm blood flow was measured for three periods, namely, at rest, during occlusion, and after occlusion, which lasted for 5 min each, and reported in the perfusion unit. Thereafter, we analyzed the resting blood flow, blood flow during occlusion, peak blood flow after occlusion, peak blood flow after occlusion/resting blood flow ratio, and recovery time after occlusion. The recovery time after occlusion referred to the after occlusion duration when the blood flow was similar to the resting value.

### 2.7 Anthropometry and body composition assessment

Anthropometry and body composition were measured when the participants were standing and wearing only the minimum necessary clothing. A body composition analyzer (InBody270, InBody Co. Ltd.) based on the principle of bioelectrical impedance analysis was used to determine body mass, body mass index, body fat percentage, fat mass, fat‐free mass, protein mass, mineral mass, and water mass. During inspiration, a stadiometer (Health‐O‐Meter ProSeries, Pelstar Inc.) was used to measure height. The mid‐point between the bottom rib and superior iliac spine was used to assess waist circumference at the end of a normal expiration. At maximum buttock extension, the hip circumference was determined. Before conducting any assessment, participants were asked to urinate. On the day before the assessments, they were also asked to refrain from eating and drinking, including alcohol and caffeine, smoking, and participating in strenuous exercise or exertion.

### 2.8 Quality of sleep assessment

The quality of sleep was assessed using the Insomnia Severity Index Questionnaire (Lukowski and Tsukerman, 2021). This questionnaire consisted of seven items in terms of sleep problems: Difficulty in falling asleep, difficulty in staying asleep, problems with waking up too early, dissatisfaction with the current sleep pattern, sleep pattern impairing the quality of life, concerns about sleep problems, and sleep problems affecting daily life.

### 2.9 Data analysis

All statistical data were analyzed using the IBM SPSS Statistics (IBM). Data normality and equal variance were studied using the Shapiro–Wilk test and Levene's test, respectively. Differences between groups before and after the intervention were assessed using the independent *t*‐test and the analysis of covariance (ANCOVA), respectively. To assess differences within a group before and after intervention, we used the paired *t*‐test. All data are presented as mean ± standard deviation. A *p* value of less than 0.05 was considered significant.

## 3 RESULTS

One person in the control group, out of 44, was excluded from the analysis due to insufficient sleep on the night before. As a result, data from 43 participants were evaluated, including 21 participants in the control group (95.45%) and 22 participants in the sleep deprivation group (100%).

### 3.1 Physical and physiological characteristics

Most of the 43 participants were female (*n* = 36, 83.72%). Sex, height, body mass, body mass index, body fat percentage, fat mass, fat‐free mass, water mass, protein mass, mineral mass, visceral fat level, metabolic rate, waist and hip circumferences, and their ratios were not significantly different between the control and sleep deprivation groups. However, age was significantly higher in the control group (*p* = 0.002) (Table 1).

**TABLE 1:** Physical and physiological characteristics of the participants

Variables	Control group (*n* = 21)	SD group (*n* = 22)	*p* value
Age (years)	22.43 ± 1.54	20.91 ± 1.23	0.002*
Sex, male/female (%)	5/16 (23.81/76.19)	2/20 (9.09/90.91)	0.413
Height (cm)	162.71 ± 7.80	162.00 ± 7.27	0.758
Body mass (kg)	52.66 ± 6.13	52.29 ± 6.21	0.847
Body mass index (kg/m^2^)	19.88 ± 1.56	20.14 ± 1.75	0.604
Body fat (%)	25.72 ± 7.61	27.78 ± 5.49	0.296
Fat mass (kg)	13.50 ± 4.24	14.38 ± 2.92	0.434
Fat‐free mass (%)	73.37 ± 8.70	71.64 ± 5.97	0.402
Fat‐free mass (kg)	38.68 ± 7.01	37.66 ± 7.06	0.808
Body water (%)	54.45 ± 5.68	52.89 ± 4.11	0.337
Water mass (kg)	28.70 ± 4.92	27.77 ± 4.97	0.544
Protein mass (kg)	7.68 ± 1.34	7.41 ± 1.33	0.504
Mineral mass (kg)	2.76 ± 0.40	2.72 ± 0.43	0.789
Visceral fat level	5.00 ± 1.90	5.50 ± 1.30	0.372
Metabolic rate (kcal/day)	1215.86 ± 143.44	1188.95 ± 145.62	0.609
Waist circumference (cm)	67.48 ± 5.53	66.16 ± 3.94	0.155
Hip circumference (cm)	89.52 ± 4.95	90.30 ± 4.88	0.317
Waist/hip circumference ratio	0.75 ± 0.05	0.73 ± 0.04	0.560

*Note*: Data are presented as mean ± standard deviation. Abbreviation: SD, Sleep deprivation.

* Significant difference between the control and sleep deprivation (SD) groups (*p* < 0.05).

### 3.2 Sleep habituation

Sleep duration was significantly lower in the sleep deprivation group than in the control group (*p* < 0.05), and habitual sleep accumulation was not significantly different between these groups (Table 2).

**TABLE 2:** Sleep habituation of the participants

Variables	Control group (*n* = 21)	SD group (*n* = 22)	*p* value
Sleep duration (h/night)	7.43 ± 0.50	6.06 ± 0.55	0.000*
Habitual sleep accumulation (months)	17.32 ± 11.33	26.32 ± 18.50	0.202

*Note*: Data are presented as mean ± standard deviation. Abbreviation: SD, Sleep deprivation.

* Significant difference between the control and sleep deprivation (SD) groups (*p* < 0.05).

### 3.3 Quality of sleep

The quality of sleep showed no significant differences between the control and sleep deprivation groups. In the analysis of all items in the quality of sleep, participants in both groups had similar sleep conditions and had no sleep problems (Table 3).

**TABLE 3:** Quality of sleep of the participants

Sleep problems	Control group (*n* = 21)	Interpretation	SD group (*n* = 22)	Interpretation	*p* value
Difficulty to falling asleep	0.64 ± 0.79	Mild	0.91 ± 0.92	Mild	0.298
Difficulty to staying asleep	0.77 ± 0.87	Mild	0.95 ± 0.79	Mild	0.471
Problems waking up too early	1.00 ± 1.15	Mild	1.5 ± 1.01	Mild	0.134
Dissatisfaction to the current sleep pattern	1.32 ± 0.78	Mild	1.23 ± 0.75	Mild	0.696
Sleep pattern impairing the quality of life	0.41 ± 0.59	Mild	0.72 ± 0.88	Mild	0.167
Concerns about sleep problems	0.68 ± 0.89	Mild	1.09 ± 1.02	Mild	0.164
Sleep problems affecting daily life	0.82 ± 0.91	Mild	1.27 ± 0.94	Mild	0.109
Summation	5.65 ± 4.13	No problem	7.73 ± 3.27	No problem	0.069

*Note*: Data are presented as mean ± standard deviation. Abbreviation: SD, Sleep deprivation.

### 3.4 Effects of sleep deprivation on FBG, endothelial function, HRV, HR, and BP variables

**FIGURE 1:**
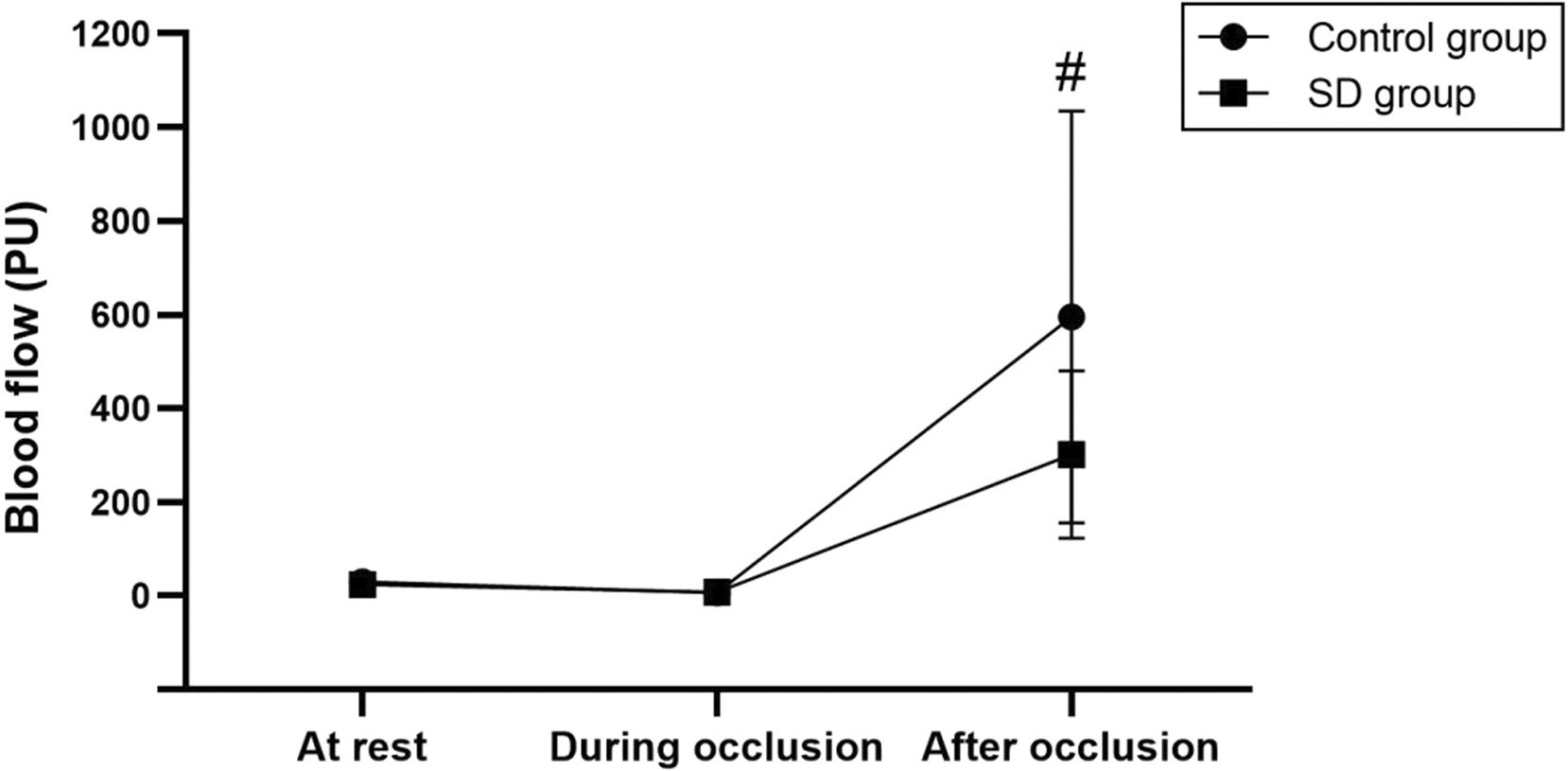
Blood flow at rest, during occlusion, and after occlusion in the control and sleep deprivation (SD) groups. Data are presented as mean ± standard deviation. ^#^Significant difference between the control and SD groups (*p* < 0.05).

The FBG level, as well as the resting blood flow and ratio of peak blood flow after occlusion/resting blood flow, were not significantly different between the control and sleep deprivation groups. However, the peak blood flow after occlusion was significantly lower (*p* < 0.05) and the recovery time after occlusion was significantly higher (*p* < 0.05) in the sleep deprivation group than in the control group (Figure 1 and Table 4).

**TABLE 4:** Fasting blood glucose and endothelial function of the participants

Variables	Control group (*n* = 21)	SD group (*n* = 22)	*p* value
Fasting blood glucose (mg/dL)	91.38 ± 6.89	89.00 ± 5.79	0.416
Endothelial function			
Resting blood flow (PU)	29.44 ± 13.05	22.91 ± 12.02	0.092
Blood flow during occlusion (PU)	5.57 ± 3.86	6.57 ± 4.30	0.429
Peak blood flow after occlusion (PU)	595.40 ± 439.63	301.30 ± 178.34	0.004*
Ratio of peak blood flow after occlusion to resting blood flow	23.91 ± 20.43	15.60 ± 11.77	0.138
Recovery time after occlusion (min)	2.11 ± 0.69	3.77 ± 1.48	<0.001*

*Note*: Data are presented as mean ± standard deviation. Abbreviations: PU, Perfusion unit; SD, Sleep deprivation.

* Significant difference between the control and sleep deprivation (SD) groups (*p* < 0.05).

**FIGURE 2:**
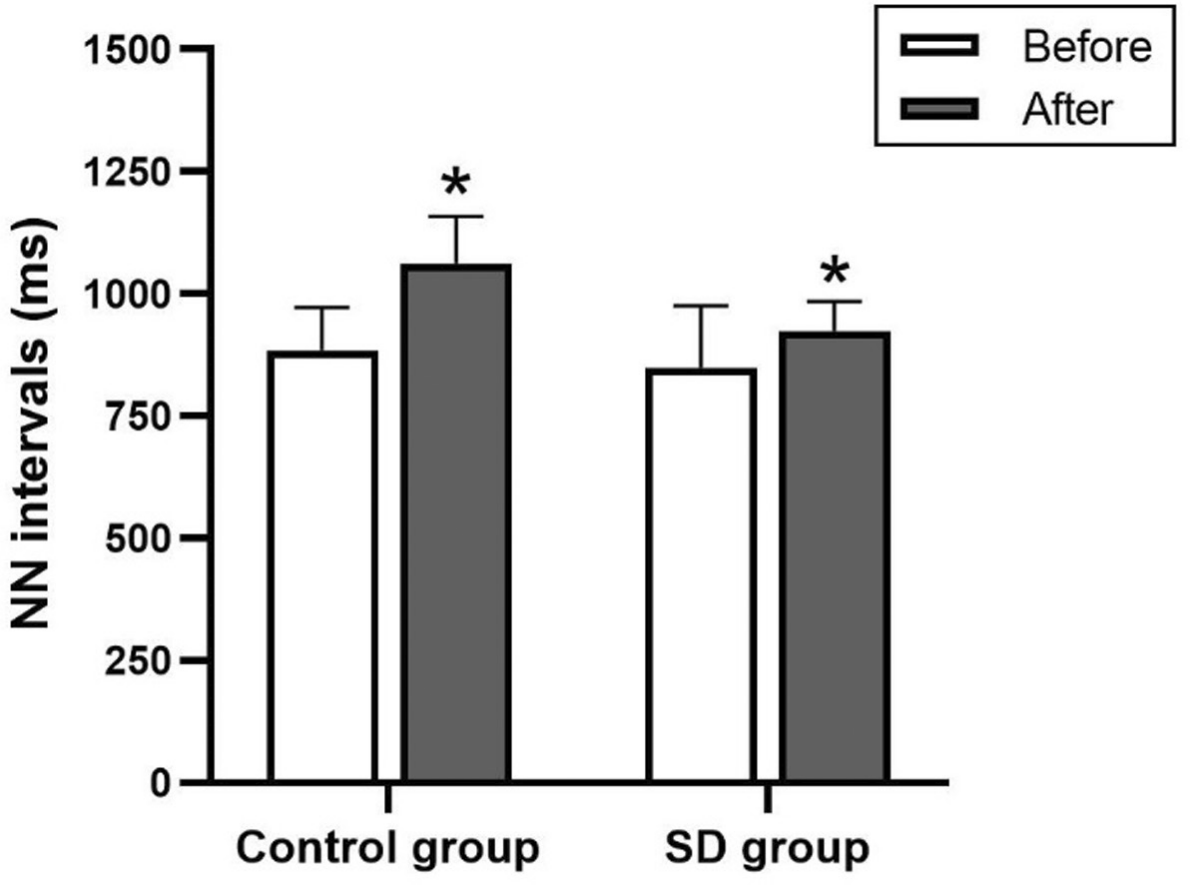
Normal‐to‐normal (NN) beat intervals in the control and sleep deprivation (SD) groups before and after 4‐7‐8 breathing control. Data are presented as mean ± standard deviation. *Significant difference compared with before 4‐7‐8 breathing control (*p* < 0.05).

**FIGURE 3:**
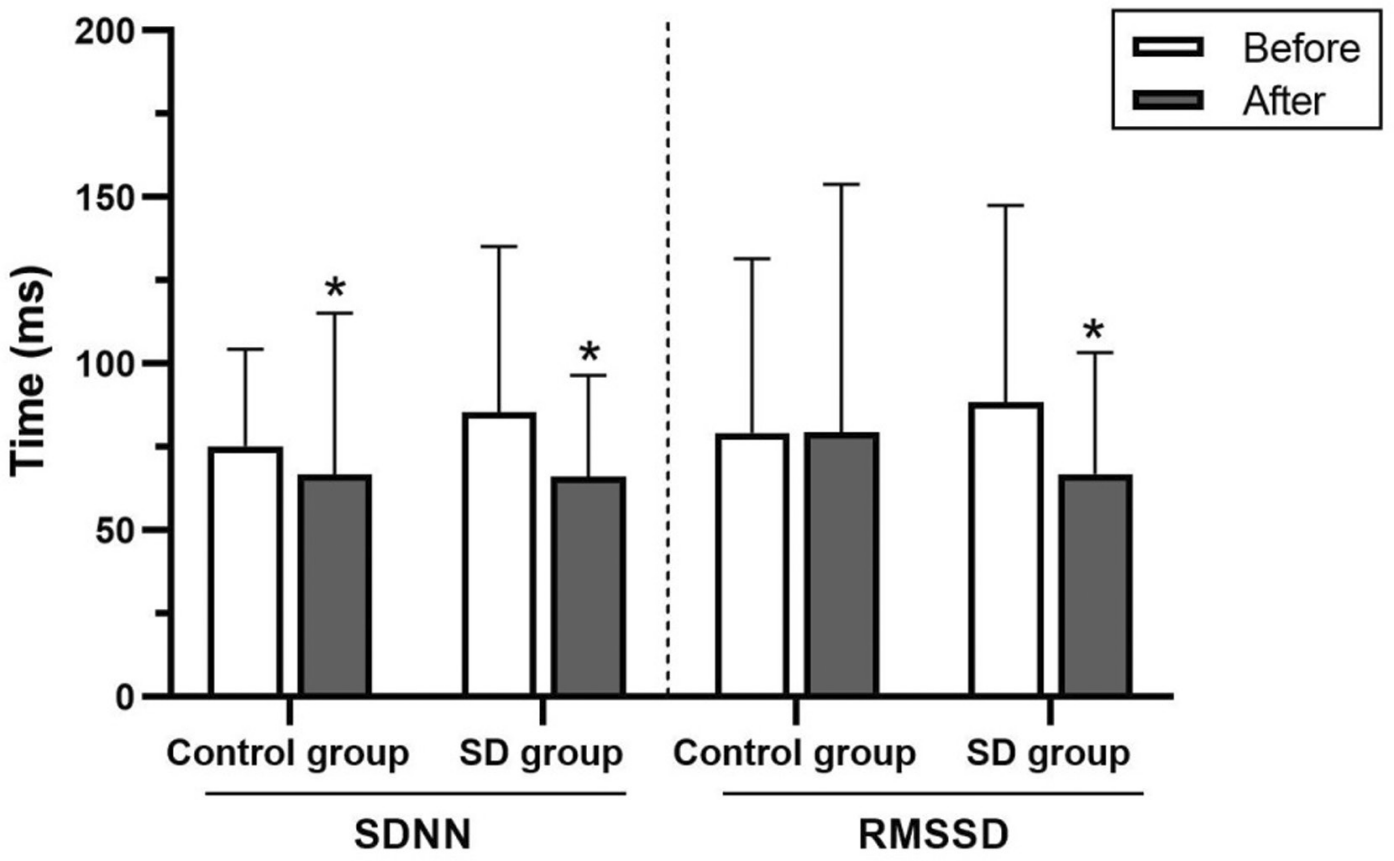
Standard deviation of normal beat‐to‐normal beat intervals (SDNN) and root mean square of successive R‐R interval differences (RMSSD) in the control and sleep deprivation (SD) groups before and after 4‐7‐8 breathing control. Data are presented as mean ± standard deviation. *Significant difference compared with before 4‐7‐8 breathing control (*p* < 0.05).

**FIGURE 4:**
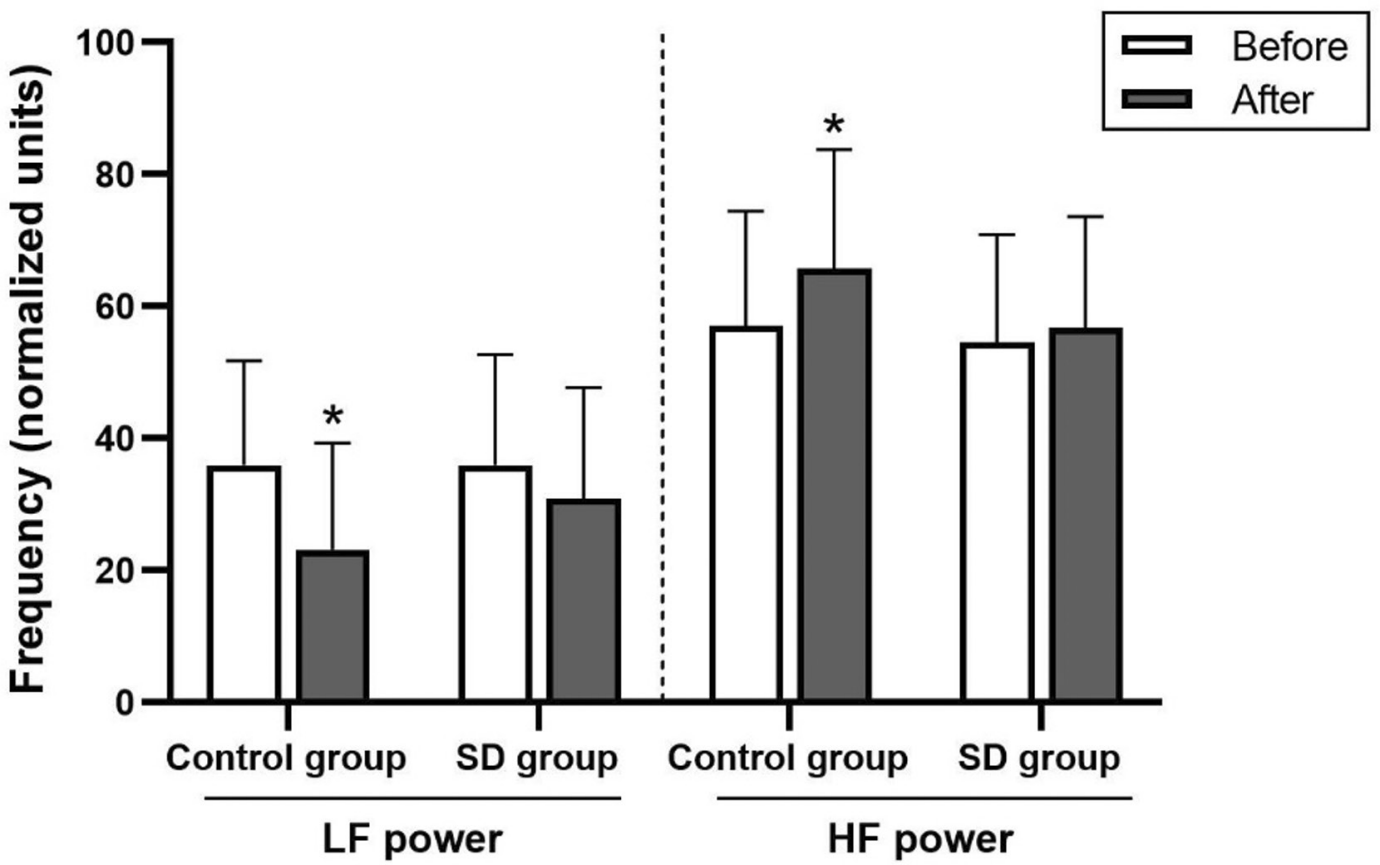
Low‐frequency (LF) and high‐frequency (HF) powers in normalized units in the control and sleep deprivation (SD) groups before and after 4‐7‐8 breathing control. Data are presented as mean ± standard deviation. *Significant difference compared with before 4‐7‐8 breathing control (*p* < 0.05).

HRV variables, including normal‐to‐normal (NN) beat intervals, SDNN, RMSSD, total power, very‐low‐frequency power, low‐frequency power, low‐frequency power in normalized units, high‐frequency power, high‐frequency power in normalized units, and low‐frequency to high‐frequency ratio, were not significantly different between the control and sleep deprivation groups (Figures 2, 3, 4 and Table 5).

**TABLE 5:** Heart rate variability of the participants before and immediately after 4‐7‐8 breathing control

Variables	Control group (*n* = 21)				SD group (*n* = 22)				*p* value (before vs. before)	*p* value (after vs. after)
	Before	After	% change	*p* value	Before	After	% change	*p* value		
NN intervals (ms)	884.92 ± 86.94	1062.37 ± 95.48	10.55	<0.001*	849.40 ± 125.92	924.22 ± 60.56	13.14	<0.001*	0.515	0.333
SDNN (ms)	75.22 ± 29.12	66.81 ± 48.42	−13.04	0.018*	85.43 ± 49.88	66.13 ± 30.44	−13.29	0.022*	0.913	0.956
RMSSD (ms)	79.30 ± 52.28	79.47 ± 74.42	2.01	0.237	88.39 ± 59.21	66.85 ± 36.49	−7.93	0.041*	0.671	0.990
Total power (ms^2^)	6289.64 ± 5462.12	5972.90 ± 10049.70	−9.62	0.012*	7779.23 ± 8275.33	4746.41 ± 4503.15	−10.68	0.020*	0.990	0.836
VLF power (ms^2^)	1676.50 ± 1233.76	603.15 ± 700.30	−58.01	<0.001*	2921.39 ± 3825.24	1087.95 ± 1139.06	−30.78	0.003*	0.568	0.112
LF power (ms^2^)	1150.47 ± 956.52	807.84 ± 1296.65	−29.71	0.018*	1430.13 ± 1437.75	1091.07 ± 1374.48	−12.07	0.221	0.743	0.388
LFnu power	35.96 ± 15.86	23.12 ± 16.13	−33.54	0.001*	35.97 ± 16.72	30.89 ± 16.80	−5.18	0.155	0.997	0.130
HF power (ms^2^)	2588.12 ± 2736.36	2202.97 ± 2703.89	−2.50	0.154	3097.27 ± 3864.65	2294.81 ± 2610.88	−42.81	0.258	0.653	0.990
HFnu power	57.03 ± 17.41	65.80 ± 18.00	21.88	0.014*	54.54 ± 16.37	56.72 ± 16.91	11.51	0.519	0.725	0.096
LF/HF ratio	0.77 ± 0.72	0.49 ± 0.70	−26.53	0.002*	0.94 ± 1.22	0.64 ± 0.48	−4.23	0.119	0.836	0.067

*Note*: Data are presented as mean ± standard deviation.Abbreviations: HF, High‐frequency; HFnu, High‐frequency in normalized units; LF, Low‐frequency; LFnu, Low‐frequency in normalized units; NN, Normal‐to‐normal beat; RMSSD, Root mean square of successive R‐R interval differences; SD, Sleep deprivation; SDNN, Standard deviation of normal beat‐to‐normal beat intervals; VLF, Very low frequency.

* Significant difference compared with before 4‐7‐8 breathing control (*P* < 0.05).

**FIGURE 5:**
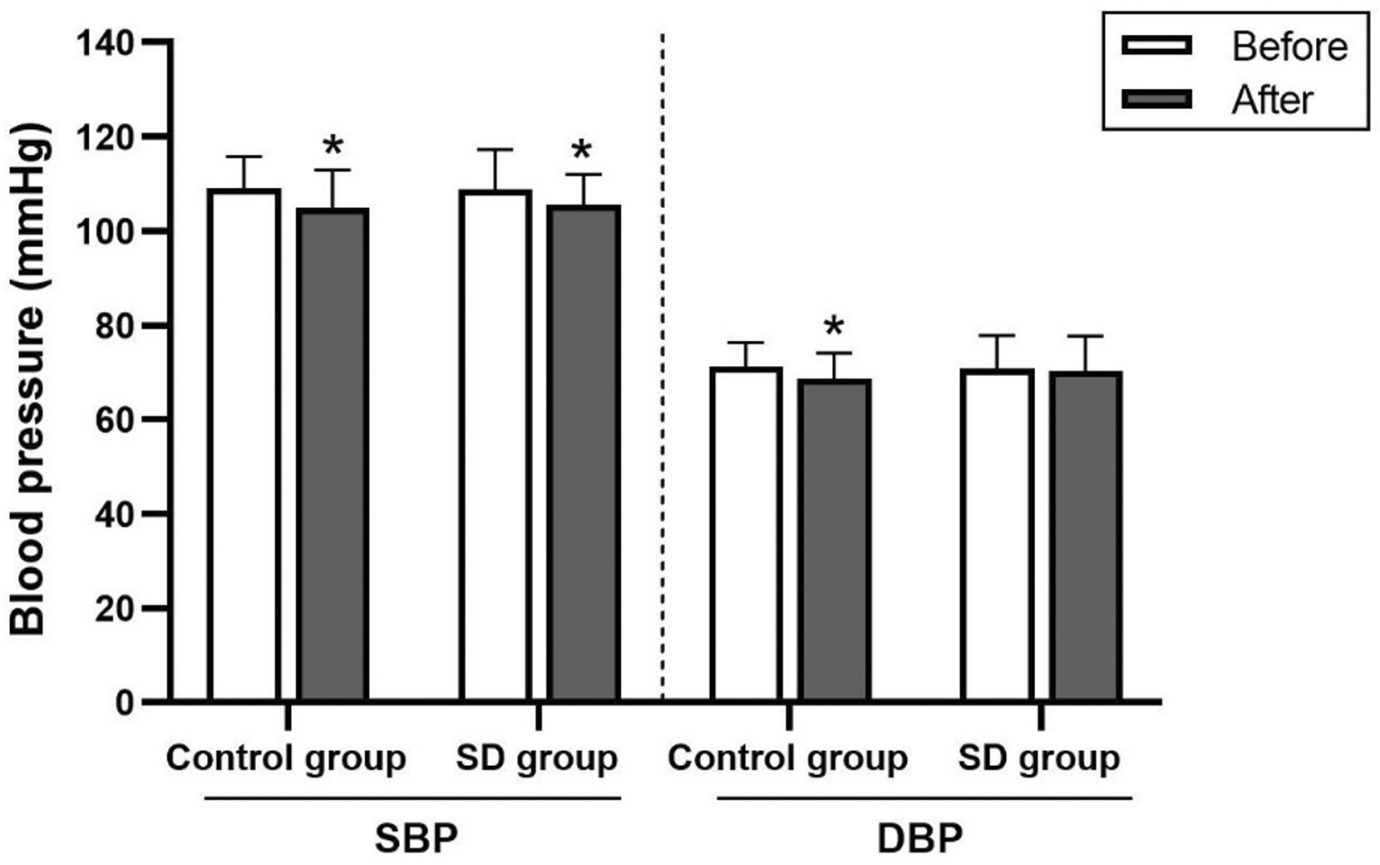
Systolic and diastolic blood pressure (SBP and DBP) in the control and sleep deprivation (SD) groups before and after 4‐7‐8 breathing control. Data are presented as mean ± standard deviation. *Significant difference compared with before 4‐7‐8 breathing control (*p* < 0.05).

Similarly, baseline HR and BP measures, such as SBP, DBP, PP, MAP, and RPP, did not differ significantly between the sleep deprivation and control groups (Figure 5 and Table 6).

**TABLE 6:** Heart rate and blood pressure of the participants before and immediately after 4‐7‐8 breathing control

Variables	Control group (*n* = 21)				SD group (*n* = 22)				*P* value (before vs. before)	*P* value (after vs. after)
	Before	After	∆ change (% change)	*p* value	Before	After	∆ change (% change)	*p* value		
HR (beats/min)	71.37 ± 7.73	66.00 ± 6.27	−5.37 (−7.21)	<0.001*	67.76 ± 11.59	64.52 ± 10.40	−3.24 (−4.51)	<0.001*	0.280	0.560
SBP (mm Hg)	109.03 ± 6.81	104.92 ± 8.02	−4.11 (−3.80)	<0.001*	108.88 ± 8.47	105.58 ± 6.45	−3.30 (−2.86)	0.003*	0.948	0.769
DBP (mm Hg)	71.35 ± 5.04	68.62 ± 5.53	−2.73 (−3.71)	0.009*	70.85 ± 7.05	70.45 ± 7.31	−0.40 (−0.50)	0.585	0.903	0.360
PP (mm Hg)	37.68 ± 5.75	36.30 ± 6.39	−1.38 (−3.69)	0.087	38.03 ± 5.94	35.12 ± 5.27	−2.91 (−7.14)	0.006*	0.846	0.512
MAP (mm Hg)	83.91 ± 5.00	80.72 ± 5.72	−3.19 (−3.77)	<0.001*	83.53 ± 7.02	82.16 ± 6.58	−1.37 (−1.54)	0.055	0.838	0.308
RPP (mm Hg bpm)	7787.62 ± 1028.35	6928.46 ± 914.21	−859.16 (−10.66)	<0.001*	7385.81 ± 1428.24	6834.14 ± 1299.41	−551.67 (−7.23)	<0.001*	0.248	0.836

*Note*: Data are presented as mean ± standard deviation. Abbreviations: DBP, diastolic blood pressure; HR, heart rate; MAP, mean arterial pressure; PP, pulse pressure; RPP, rate‐pressure product; SBP, systolic blood pressure; SD, sleep deprivation.

* Significant difference compared with before 4‐7‐8 breathing control (*p* < 0.05).

### 3.5 Effects of 4‐7‐8 breathing control on HRV, HR, and BP variables

In response to the 4‐7‐8 breathing control, SDNN, total power, very‐low‐frequency power, low‐frequency power, low‐frequency power in normalized units, and low‐frequency to high‐frequency ratio significantly decreased (*p* < 0.05), whereas, NN intervals and high‐frequency power in normalized units significantly increased in the control group (*p* < 0.05). In the sleep deprivation group, SDNN, RMSSD, total power, and very‐low‐frequency power were significantly reduced (*p* < 0.05) and NN intervals significantly increased (*p* < 0.05). However, the HRV variables showed no significant between‐group differences (Figures 2, 3, 4 and Table 5). In addition, there were no significant between‐group differences in terms of percentage change.

Moreover, the control group had significantly decreased HR, SBP, DBP, MAP, and RPP levels (*p* < 0.05). Similarly, the sleep deprivation group had significantly decreased HR, SBP, PP, and RPP levels (*p* < 0.05). However, the HR and BP variables revealed no significant between‐group differences (Figure 5 and Table 6). Moreover, there were no significant between‐group differences in terms of delta and percentage changes.

## 4 DISCUSSION

### 4.1 Effects of sleep deprivation on FBG, endothelial function, HRV, HR, and BP variables

FBG levels and HRV, HR, and BP variables in participants with and without sleep deprivation were not significantly different in this study. Despite this, participants who were sleep deprived had significantly lower endothelial function.

FBG data suggest that factors other than sleep duration were involved in regulating FBG levels. Although sleep duration was statistically lower in the sleep deprivation group than that in the control group and sleep guidelines are well‐established, this distinction may not be clinically sufficient. Moreover, the quality of sleep—which is highly important—and the habitual sleep accumulation did not differ between the two groups. These factors may play an important role in concealing a significant difference in this study, which explored healthy and young individuals. In addition, as most of our participants were female, gonadal hormone—estrogen—may have affected FBG levels. As reported in earlier studies, females have lower FBG levels and higher insulin sensitivity than males, suggesting greater glucose homeostasis in premenopausal females (Mauvais‐Jarvis, 2018). Importantly, this study did not compare FBG levels to insulin levels or insulin sensitivity. This constraint may make fully mechanistic explanations difficult. In contrast to our findings, another study found that healthy young males with chronic sleep deprivation (4 h/night) had reduced glucose tolerance (Spiegel et al., 1999). Similarly, healthy males who were exposed to a single night of sleep restriction (4 h/night) had reduced insulin sensitivity (Sweeney et al., 2020).

The participants in the sleep deprivation group had a lower peak blood flow after occlusion release and a higher recovery time after occlusion than those in the control group. Thus, vasodilation and vascular adaptation efficiency in response to occlusion may be impaired in participants with sleep deprivation. Our findings are consistent with those of Cherubini et al. (2021), who found that short sleep durations decrease endothelial function and alter autonomic balance as well as the circadian rhythmicity of peripheral vascular clock components. This study also found an association between short sleep duration and endothelial dysfunction, which leads to morbidity. Similarly, Holmer et al. (2021) found that sleep deprivation is related to decreased macrovascular endothelial function and impaired microvascular endothelial function in a recent systematic review.

HRV, HR, and BP data suggest that these variables were influenced by several factors. Although sleep duration was shorter in the sleep deprivation group than in the control group, other important determinants, including quality of sleep and habitual sleep accumulation, were not different. Furthermore, the difference in sleep durations between the two groups (sleep deprivation group: 6.06 ± 0.55 h versus control group: 7.43 ± 0.50 h) in this study is smaller than that in Zhang et al.'s CONSORT study (2021) (sleep deprivation group: 3.78 ± 0.69 h versus control group: 7.63 ± 0.52 h). According to our study results, although participants with sleep deprivation may have impaired endothelial function, similarity in HRV, HR, and BP variables may indicate that their cardiovascular systems can adapt well to such physiological and psychological stresses (van Leeuwen et al., 2018). Cardiac autonomic function and BP may also vary according to sex. Females have better HRV than males, with increased parasympathetic tone, attributed inherently to the hormonal difference between the two sexes. Explicitly, estrogen increases the parasympathetic tone, whereas testosterone increases the sympathetic tone (Punita et al., 2016). Furthermore, premenopausal females often have a lower BP than males of the same age. Estrogens and chromosomes are likely to have diverse effects on BP control mechanisms (Maranon and Reckelhoff, 2013). However, in a CONSORT study by Zhang et al. (2021), healthy participants with sleep deprivation had lower cardiac vagal activity, as evidenced by lower RMSSD, high‐frequency power in normalized units, SD1, and pNN50 values, than those without sleep deprivation. Furthermore, Li et al. (2019) reported that those who slept for less than 7 h per day had a higher risk of hypertension than individuals who slept 7–8 h per day in a large‐scale study (19,407 adults aged 18–79 years). Additionally, in females with sleep duration reduced by 1.5 h/night (sleep approximately 6.17 h/night) for 6 weeks, DBP and MAP were higher than those in females with usual sleep duration (St‐Onge et al., 2020).

### 4.2 Effects of 4‐7‐8 breathing control on HRV, HR, and BP variables

After the 4‐7‐8 breathing control, HRV measures, such as SDNN, total power, and very‐low‐frequency power, significantly decreased, whereas NN intervals significantly increased in participants with and without sleep loss. Furthermore, HR and BP indicators, such as SBP and RPP, decreased significantly in both participant groups. However, no significant differences were found between the two groups.

In practicing the 4‐7‐8 breathing control (inhale:exhale ratio = 1:2), participants' respiratory rate was approximately 3 breaths/min. This breathing pattern is similar to deep and slow breathing, which has previously been investigated. Magnon et al. (2021) conducted a study in young and older healthy volunteers performing deep and slow breathing (low inhale/exhale ratio) and found that high‐frequency power was significantly increased in both groups, indicating the restoration of vagal outflow after the deep and slow breathing. Our recent study, which examined immediate alterations in HRV in overweight and obese adults, demonstrated that the NN intervals, RMSSD, SD2, high‐frequency power, and low‐frequency power were significantly greater in the slow diaphragmatic breathing control group than in the spontaneous breathing group; the SBP, DBP, and MAP also significantly decreased in the diaphragmatic breathing control group (Khamsuk et al., 2021). These data could confirm that slow breathing control enhances parasympathetic activity and lowers BP. However, Chinagudi et al. (2014) demonstrated that in healthy adults with sympathetic predominance, high‐frequency power in normalized units significantly decreased, whereas low‐frequency power in normalized units and low‐ to high‐frequency ratio significantly increased after slow and deep breathing at 6 breaths/min for 5 min. However, in their study, the inhale/exhale ratio was 1.

The aforementioned data suggest that parasympathetic activity is augmented by breathing with a low inhale/exhale ratio. Moreover, additional holding of one's breath during inhalation can increase arterial oxygen saturation and subsequently decrease peripheral chemoreceptor stimulation, thereby enhancing the parasympathetic activity and lowering the BP (Turankar et al., 2013; Williams et al., 2019). Notably, our study revealed that in the control group, significant decreases in low‐frequency power, low‐frequency power in normalized units, and low‐frequency to high‐frequency ratio following the 4‐7‐8 breathing control indicate less sympathetic activity. A significant decrease in low‐frequency to high‐frequency ratio and a significant increase in high‐frequency in normalized units further indicate greater respiratory sinus arrhythmia (RSA) and parasympathetic activity. RSA, which is HRV in synchrony with respiration, is an index of cardiac vagal function and reflects respiratory‐circulatory interactions. With greater RSA, gas exchange in alveoli may be enhanced by accumulating ventilation/perfusion matching efficiency (Yasuma and Hayano, 2004).

In this study, very‐low‐frequency power significantly decreased in both the control and sleep deprivation groups. Although very‐low‐frequency power is not exactly known, it may be generated after exposure to mental stress (Usui and Nishida, 2017). When considering the 4‐7‐8 breathing control intervention, our participants might partially encounter mental stress because they had been unfamiliar with this practice. Here, the participants were not doing any kind of meditation or breathing control before the study.

Total power is the sum of the powers of the five frequencies, and the very‐low‐frequency and ultra‐low‐frequency powers account for most of the total power (Yılmaz et al., 2018). Thus, a decrease in very‐low‐frequency power caused the decreased total power in both the control and sleep deprivation groups. SDNN is produced from both sympathetic and parasympathetic activities and is highly related to ultra‐low‐frequency, very‐low‐frequency, and low‐frequency powers, and total power. Accordingly, the decreased SDNN in both the control and sleep deprivation groups may be caused by a reduction in sympathetic activity or a lessening in very‐low‐frequency power or total power.

This study also observed that RMSSD significantly decreased in the sleep deprivation group. RMSSD, which is the principal time domain of HRV, reflects the parasympathetic activity and is associated with high‐frequency power (Shaffer and Ginsberg, 2017). Our results are consistent with the findings of a study by Chinagudi et al. (2014), who reported that after slow and deep breathing at 6 breaths/min (inhale:exhale = 1:1), RMSSD decreased (not significant). As the participants were experiencing slow deep breathing exercises for the first time, their sympathetic system's fight‐or‐flight response may have been activated. However, the sympathetic activity reflected by low‐frequency power did not increase in our study. In fact, RMSSD decrease was observed only in the sleep deprivation group. Thus, the decreased RMSSD may indicate a potential deterioration in parasympathetic tone in response to physiological stimuli in persons with sleep deprivation (Bourdillon et al., 2021). This indication is supported by a decremental change observed in the high‐frequency power that was greater (not significant) in the sleep deprivation group (−42.81%) than that in the control group (−2.50%).

We also found that the 4‐7‐8 breathing control decreased the HR and BP variables in almost all participants in both the control and sleep deprivation groups. The results of this study are consistent with previous findings (Mitsungnern et al., 2021; Pramanik et al., 2010; Tripathy et al., 2019). In patients with hypertensive urgency, SBP, DBP, and HR values were significantly lower than the baseline values after deep/slow breathing, and these reductions were greater than those in the control group without deep/slow breathing (Mitsungnern et al., 2021). Moreover, Pramanik et al. (2010) reported that SBP, DBP, MAP, and HR values decreased in volunteers after slow paced breathing for 5 min. Additionally, a study by Tripathy et al. (2019) in male yogic practitioners demonstrated that SBP, DBP, and HR values significantly decreased after performing Nadi Shodhana pranayama for 20 min. They suggested that practicing deep inhalation and slow exhalation improves supplemental oxygen supply throughout the body, activates the parasympathetic nervous system, and improves healthy cardiovascular function.

### 4.3 Strengths and limitations of the study

To the best of our knowledge, this is the first study evaluating the immediate effects of 4‐7‐8 breathing control on HRV and BP in people with sleep loss. However, there are several limitations to this research. The small sample size of males in this study appears to be insufficient to accurately reflect the general population's condition. As sex hormones can affect cardiac autonomic function, BP, and FBG, the results could have been influenced by the high female‐to‐male ratio. Furthermore, after the 4‐7‐8 breathing control, endothelial function was not evaluated. Hence, although the intervention's magnitude may not have been sufficiently high to affect endothelial function, this study may not provide complete data, and further research is needed. This study investigated the immediate effects of the 4‐7‐8 breathing control in people with sleep deprivation. Therefore, future research should investigate the long‐term effects of this breathing practice on cardiovascular and cardiac autonomic functioning over a period of months or years. It should also investigate the efficacy of 4‐7‐8 breathing control intervention in other populations, such as older people who may have sleep issues, patients with respiratory disorders who want to improve perfusion and gas exchange, and patients with mental illnesses who want to improve cognition, emotion, and behavior.

## 5 CONCLUSIONS

Healthy young adults with sleep deprivation and adequate sleep demonstrated similar HRV, BP, and FBG. Nevertheless, endothelial function may be lower in those with sleep deprivation. Furthermore, by increasing parasympathetic activity and decreasing sympathetic activity, the 4‐7‐8 breathing control may improve HRV and BP, especially in people without sleep deprivation. This intervention may also be beneficial for patients with cardiovascular disease or pulmonary disease in terms of reducing cardiac work and enhancing blood oxygenation.

## AUTHOR CONTRIBUTIONS

Piyapong Prasertsri designed the study. Jaruwan Vierra performed data collection. Piyapong Prasertsri performed statistical analysis and interpreted the data. Jaruwan Vierra, Orachorn Boonla, and Piyapong Prasertsri contributed to writing and proofing of the final version of the manuscript.

## CONFLICT OF INTEREST

No conflicts of interest are reported by the authors of this paper.
